# Usefulness of the maximum rate of pressure rise in the central and peripheral arteries after weaning from cardiopulmonary bypass in pediatric congenital heart surgery: A retrospective analysis

**DOI:** 10.1097/MD.0000000000005405

**Published:** 2016-12-09

**Authors:** Jung-Won Kim, Ji-Yeon Bang, Chun Soo Park, Mijeung Gwak, Won-Jung Shin, Gyu-Sam Hwang

**Affiliations:** a Department of Anesthesiology and Pain Medicine, Catholic Kwandong University International St. Mary's Hospital, Incheon; b Department of Anesthesiology and Pain Medicine; c Laboratory for Cardiovascular Dynamics, Asan Medical Center, University of Ulsan College of Medicine; d Division of Pediatric Cardiac Surgery, Asan Medical Center, University of Ulsan College of Medicine, Seoul, Korea.

**Keywords:** aortic maximum rate of pressure rise, congenital heart surgery, postoperative ventricular function, radial maximum rate of pressure rise

## Abstract

The maximum rate of pressure rise (dP/dt_max_) in radial artery has been proposed as a noninvasive surrogate of aortic dp/dt_max_, reflecting left ventricular (LV) contractility in children. The aim of this study was to investigate relationship between aortic and radial dp/dt_max_ at weaning from cardiopulmonary bypass (CPB) and usefulness of these indices for estimating postoperative outcomes in pediatric congenital heart surgery.

Aortic and radial arterial pressure waveforms were analyzed simultaneously during weaning from CPB in 29 congenital heart surgery. The maximum first derivatives of aortic and radial arterial waveforms were calculated and averaged from 3 consecutive respiratory cycles. We obtained the maximum vasoactive inotropic score during the first 36 postoperative hours, LV ejection fraction, and fractional shortening on transthoracic echocardiography performed within postoperative day 7.

A significant difference between aortic and radial dP/dt_max_ was observed (mean difference 356 mm Hg/s, 44% of averages), and radial dP/dt_max_ was weakly correlated with aortic dP/dt_max_ (*r* =0.373, *P* = 0.047). Aortic dP/dt_max_ was significantly associated with the maximum vasoactive inotropic score (*P* *<* 0.001), postoperative LV ejection fraction (*P* = 0.018), and fractional shortening (*P* = 0.015); however, radial dP/dt_max_ was not. On Receiver operating characteristic analysis, aortic dP/dt_max_ had a greater area under the curve than radial dP/dt_max_ in predicting higher vasoactive inotropic score (0.827 vs 0.673).

Immediately after CPB in pediatric congenital heart surgery, radial dP/dt_max_ may not replace aortic dP/dt_max_ because of a discrepancy between central and peripheral arterial waveforms. In this critical period, aortic dP/dt_max_ can be useful to estimate postoperative ventricular function rather than peripherally derived dP/dt_max_.

## 1 Introduction

The maximum rate of the left ventricular pressure rise (LV dP/dt_max_) has been known to reflect the LV contractility, which is not influenced by wall motion abnormalities, afterload or variations in ventricular anatomy and morphology.^[1–4]^ However, the measurement of LV dP/dt_max_ is undoubtedly invasive monitoring, which is required intraventricular catheterization, which is limited as a bedside clinical feasibility particularly in small children.

As a less invasive index, it has been suggested that the maximum rate of aortic pressure rise (Ao dP/dt_max_) can accurately predict the LV dP/dt_max_ in pediatric patients with congenital and acquired cardiac disease.^[5]^ Recently, the dp/dt_max_ tonometrically measured at the brachial and radial arteries has also been found to show excellent correlation with the LV dP/dt_max_ in children underwent cardiac catheterization for various cardiovascular abnormalities.^[6]^ Therefore, the peripheral dP/dt_max_ derived from pressure waveform analysis seems to be useful to assess ventricular contractility as a noninvasive intraoperative monitoring, particularly in pediatric cardiac surgery.

However, immediately after patients are weaned from CPB, there is a discrepancy in the pressure waveform between central and peripheral arteries because of changes in vascular characteristics.^[7,8]^ Moreover, the relationship between the Ao dP/dt_max_ and peripheral arterial dP/dt_max_ during cardiac surgery in children remains uncertain, especially during the period of weaning from CPB. Therefore, we investigated the relationship between the aortic and radial dP/dt_max_ immediately after weaning from CPB in pediatric congenital heart surgery. In addition, we also evaluated whether these parameters are associated with immediate postoperative LV function.

## 2 Materials and methods

### 2.1 Study population

This retrospective analysis of prospectively collected data was approved by the Institutional Review Board of Asan Medical Center, Seoul, Korea (No 2014-0773). We investigated the electrical medical records of 34 consecutive children who underwent corrective cardiac surgery at our single tertiary care hospital between January 2013 and February 2014 and who were assigned to Risk Adjustment in Congenital Heart Surgery (RACHS-1) category 1–2.^[9]^ All operations were performed by the same pediatric cardiac surgeon (C.-S. Park). We excluded 5 patients who did not require CPB, incomplete data collection, or poor signal quality of arterial waveforms, and thus 29 patients were available for final analysis.

### 2.2 General anesthesia and clinical practice

Patients arrived at the operating room lightly sedated following premedication with 0.05 to 0.075 mg/kg of intravenous midazolam. Patients received routine monitoring using 5-lead electrocardiography, pulse oximetry, noninvasive blood pressure, and capnography. In accordance with the standard protocol at our institution,^[10,11]^ anesthesia was induced with midazolam (0.1–0.2 mg/kg), thiopental sodium (1.5–2.0 mg/kg), or ketamine (1–2 mg/kg), as appropriate. After the patient received vecuronium (0.15 mg/kg) and fentanyl (1–3 μg/kg), tracheal intubation was performed. Anesthesia was maintained with boluses of midazolam (0.1–0.2 mg/kg), rocuronium (0.5 mg/kg), and fentanyl (3–5 μg/kg) given every 30 to 40 minutes. Direct arterial catheterization (24 G; Becton Dickinson Infusion Therapy Systems Inc., Sandy, Utah) of the radial artery was performed to continuously monitor the arterial pressure. The central venous pressure was also monitored using ultrasound-guided placement of a pediatric multi-lumen central venous catheter (4–5.5 Fr; Arrow International Inc., Reading, PA). Cerebral oxygen saturation was also continuously monitored using near-infrared spectroscopy (INVOS 5100, Somanetics, Troy, MI). CPB was started with aortic cannulation (Medtronic, Minneapolis, MN) and bicaval venous drainage, and systemic cooling (25–28°C) was obtained uniformly under 2.4 L/min/m^2^ of the perfusion index. Modified ultrafiltration was used for all patients immediately after separation from CPB. To achieve hemodynamic stability, dopamine (3–10 μg/kg/min), milrinone (0.375–0.75 μg/kg/min), or epinephrine (0.02–0.1 μg/kg/min) were infused continuously, as appropriate. After CPB, transfusion of filtered red blood cells was used to maintain the hematocrit greater than 30%. Before aortic decannulation, protamine (1 mg/100 units of heparin) was administered to reverse heparin.

### 2.3 Hemodynamic variables and measurements of the Ao dP/dt_max_ and radial dP/dt_max_

**Figure 1:**
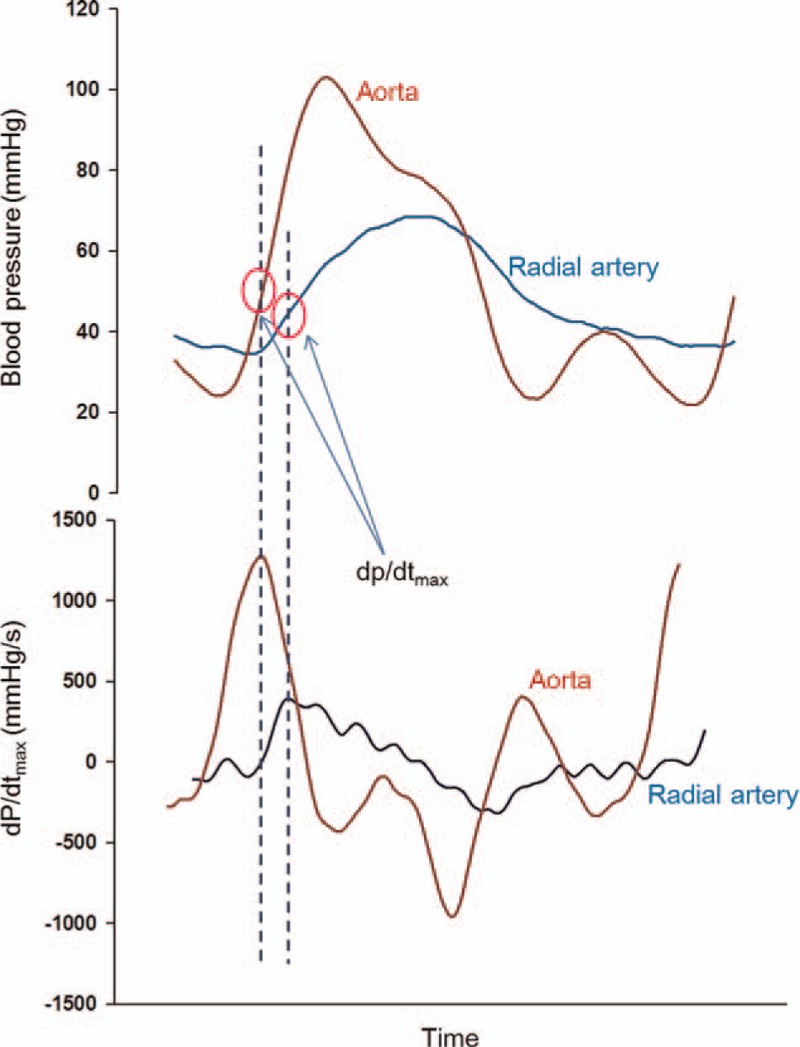
Example tracings of arterial pressure waveforms (upper panel) and the first derivatives (dP/dt) recorded at aorta and radial artery. The circle indicates the measuring point of dP/dt_max_.

Baseline systolic and diastolic noninvasive blood pressure (SBP and DBP), heart rate and arterial oxygen saturation (SpO_2_) were recorded before anesthesia. Following anesthetic induction, we also obtained pre-CPB hemodynamic variables; radial SBP and DBP, heart rate, SpO_2_, radial dP/dt_max_ and cerebral oxygen saturation. After completion of CPB, a pressure monitoring line was connected into the ascending aorta cannula to continuously measure the aortic pressure. Data on the continuous arterial pressure waveform of the ascending aorta and radial artery were simultaneously collected using a personal computer interfaced with a Windaq analog/digital converter (DATAQ Instruments Inc., Akron, OH). For each patient, aortic and radial artery pressure waveforms were abstracted during the first 5-minute period immediately after cessation of CPB. The Ao dP/dt_max_ and Radial dP/dt_max_ were obtained by calculation of the maximum first derivatives of the aortic and radial arterial pressure waveforms using a signal processing software program (CODAS, DATAQ; DADiSP/Adv DSP; DSP Development, Cambridge, MA) (Fig. 1). We also recorded aortic and radial SBP and DBP, and all pressure-derived data were averaged for 3 consecutive respiratory cycles.

### 2.4 Assessment of early postoperative LV function and outcomes

To quantify the requirement for inotropic and vasoactive drugs, we calculated the maximum vasoactive inotropic score (VIS_max_) for each patient during the first 36 hours after surgery.^[12,13]^ The inotropic score was calculated as follows: (dopamine [μg/kg/min]) + (dobutamine [μg/kg/min]) + (10000 × vasopressin [U/kg/min]) + (10 × milrinone [μg/kg/min]) + (100 × epinephrine [μg/kg/min]) + (100 × norepinephrine [μg/kg/min]).^[12]^ Transthoracic echocardiography was performed in all patients within 7 postoperative days before discharge from the hospital. The 2D- echocardiographic images from the apical 4- and 2-chamber views showing the LV measurements were used with the modified Simpson's method to estimate the LV ejection fraction (EF). LV fractional shortening (FS) was measured from an M-mode trace in the parasternal long-axis view. We also obtained outcome data, including the need for inhaled nitric oxide, duration of mechanical ventilation, and length of postoperative hospital stay.

### 2.5 Statistical analysis

Data are expressed as mean with standard deviation or median with the interquartile range, as appropriate. The Bland–Altman method was used to assess of agreement between variables derived from aortic and radial arterial pressure waveforms. The correlation between the Ao dP/dt_max_ and radial dP/dt_max_ was tested with Pearson's correlation coefficient. We also performed Spearman's rank correlation between dP/dt_max_ and the early postoperative outcomes. Receiver operating characteristics (ROC) analysis was used to assess the ability of the dp/dt_max_ of the aorta and radial artery to predict decreased ventricular function (EF <50% and FS<24%), greater requirement of inotropic and vasoactive drugs, prolonged mechanical ventilation, or postoperative hospital stay, defined as upper quartile in distribution. We calculated assuming that the area under the ROC curve representing the ability of dP/dt_max_ to determine the postoperative outcome was 0.8, and that correlation coefficient between aortic and radial dP/dt_max_ was 0.5. It would be required to include 28 patients, based on an α significance of 0.05 and a β error of 0.2. A *P*-value less than or equal to 0.05 was considered significant. Analyses were performed using SPSS version 21.0 software (IBM SPSS Inc., Chicago, IL) and SigmaPlot version 12.0 (Systat Inc., San Jose, CA).

## 3 Results

Twenty-nine patients were included in final analyses. Five patients were excluded because they had an incomplete continuous arterial waveform, such as missing data or poor signal quality. None of the 29 children in our current study had a residual shunt or died during their period of hospitalization. Eighteen of these patients had a ventricular septal defect, 8 were had an atrial septal defect, 1 had ventricular septal defect with concomitant pulmonary stenosis, and 2 had Tetralogy of Fallot. Patient demographic data and perioperative clinical variables are summarized in Table 1.

**Table 1:**
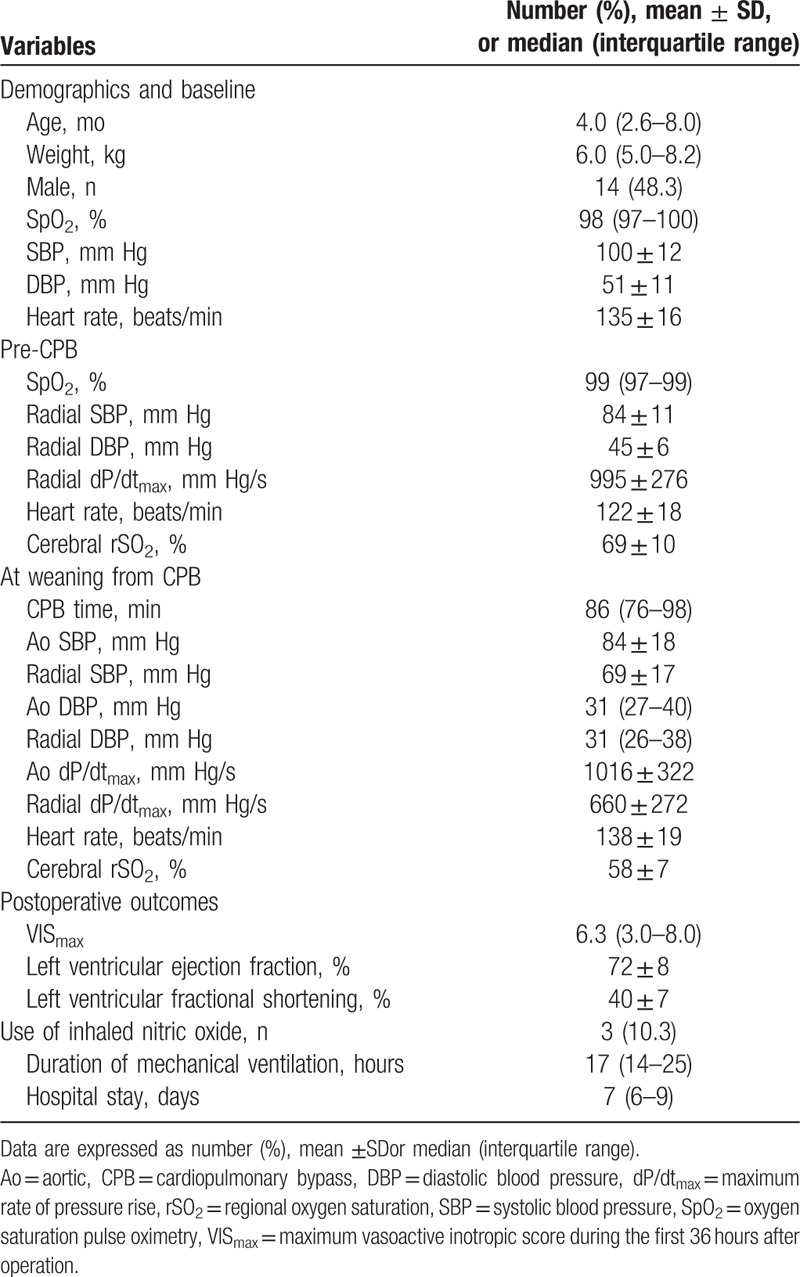
Patient demographic data and perioperative clinical variables.

**Figure 2:**
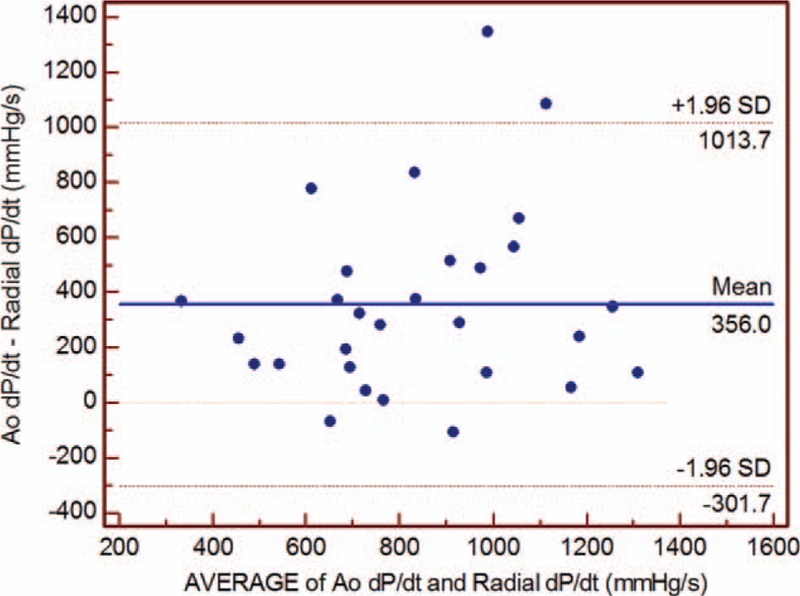
The Bland–Altman plot for the maximum pressure rise in the aorta (Ao dP/dt_max_) and radial artery (radial dP/dt_max_). Ao = aortic, dP/dt_max_ = maximum rate of pressure rise.

**Figure 3:**
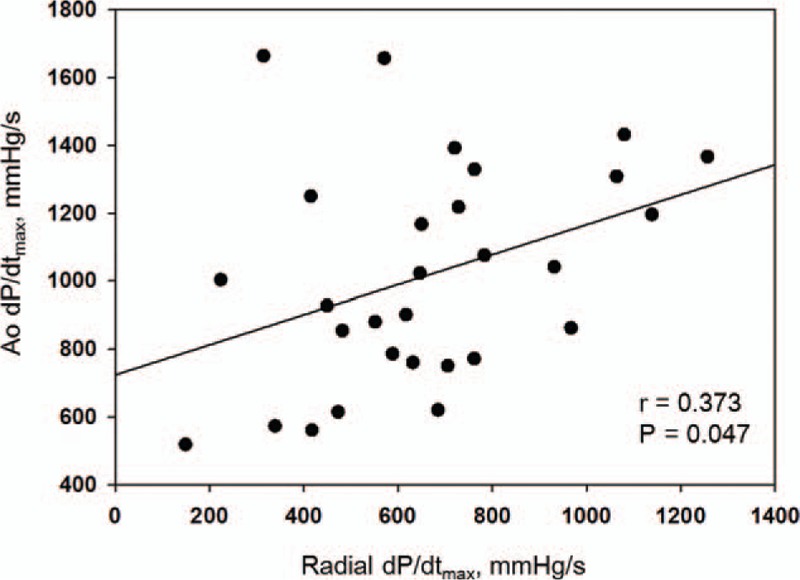
Correlation between the maximum pressure rise in the aorta (Ao dP/dt_max_) and radial artery (radial dP/dt_max_). Ao = aortic, dP/dt_max_ = maximum rate of pressure rise.

Immediately after weaning from CPB, radial SBP showed lower value than aortic SBP (mean difference 15.6 mm Hg; 95% limits of agreement –6.9, 38.1), whereas DBP was similar between aorta and radial artery (mean difference; 95% limits of agreement –6.1, 8.1) (Table 1). The mean difference was 356 mm Hg/s (44% of averages) between Ao and radial dP/dt_max_ (Fig. 2). The radial dP/dt_max_ weakly but significantly correlated with the Ao dp/dt_max_ immediately after CPB weaning (*r* = 0.373, *P* = 0.047; Fig. 3).

**Figure 4:**
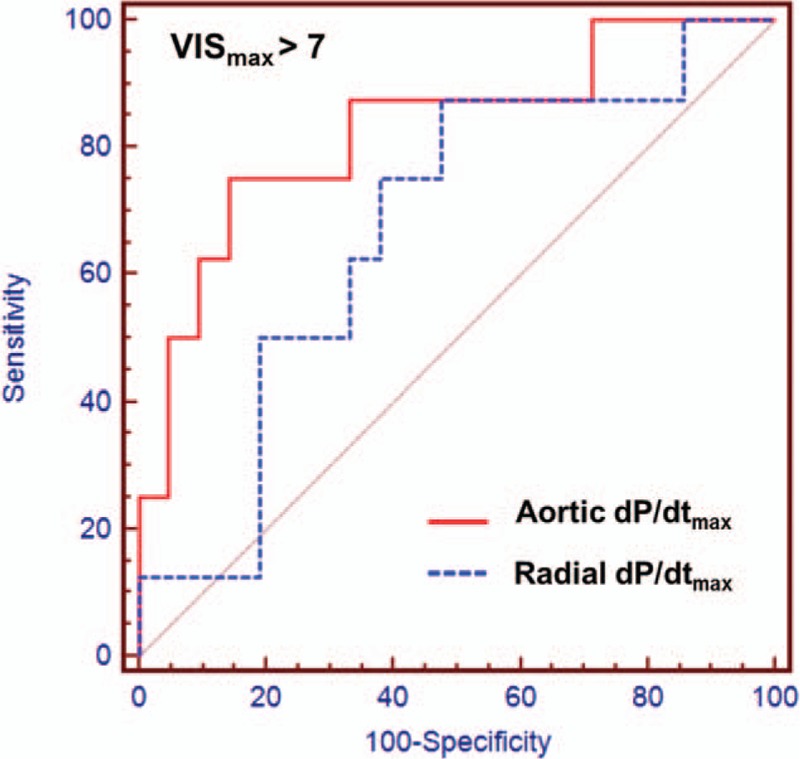
Receiver operating characteristic curves comparing the maximum pressure rise in the aorta (Ao dP/dt_max_) and radial artery (radial dP/dt_max_) to predict the maximum vasoactive inotropic score (VIS_max_) >7. The area under the curve of Ao dP/dt_max_ (0.827) is higher than that of radial dP/dt_max_ (0.673). Ao = aortic, dP/dt_max_ = maximum rate of pressure rise, VIS_max_ = maximum vasoactive inotropic score.

There was an inverse correlation between the Ao dP/dt_max_ and requirement for postoperative vasoactive inotropic use (*r* = −0.625, *P* < 0.001). The radial dP/dt_max_ was negatively associated with the postoperative VIS_max_, but statistically insignificant (*r* = −0.366, *P* *=* 0.051). The Ao dP/dt_max_ also significantly correlated with the postoperative LVEF (*r* = 0.453, *P* = 0.018) and LVFS (*r* = 0.470, *P* = 0.015), unlike the radial dP/dt_max_ (*P* = 0.118 and *P* = 0.126, respectively). The duration of mechanical ventilation, length of postoperative hospital stay, and need for postoperative inhaled nitric oxide did not significantly correlate with the Ao dP/dt_max_ or radial dP/dt_max_. In ROC curve analysis, the area under the curve (AUC) of the Ao dP/dt_max_, predicting high requirement of vasoactive inotropic drugs was greater than the AUC of the radial dP/dt_max_ (Table 2, Fig. 4). A post-CPB Ao dP/dt_max_ ≤ 785 mm Hg/s could discriminate VIS_max_ greater than 7 (sensitivity, 75.0%; specificity, 85.7%). We also observed that the Ao dP/dt_max_ also had greater AUCs to predict decreased LV function and prolonged mechanical ventilation than those of the radial dP/dt_max_ (Table 2).

**Table 2:**
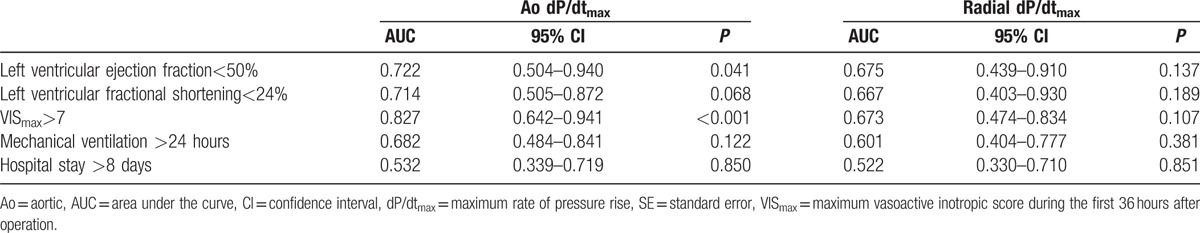
Receiver operating characteristic curves of aortic and radial dP/dtmax to predict poor postoperative outcomes.

## 4 Discussion

Immediately after weaning from CPB in pediatric congenital heart surgery, this study showed that the radial dP/dt_max_ was consistently lower than the Ao dP/dt_max_, and weakly correlated with the Ao dP/dt_max_. In addition, the Ao dP/dt_max_ had higher AUC for predicting decrease in LVEF and LVFS, greater requirement of vasoactive inotropic drugs, prolonged mechanical ventilation, and postoperative hospital stay than the radial dP/dt_max_ during this period.

Children with congenital heart disease have distorted ventricular geometry and volume- or pressure-overloaded ventricles. Therefore, it has been known to be difficult to assess ventricular contractility using the conventional echocardiographic technique.^[14,15]^ The LV dP/dt_max_ may be reliable and accurate to quantify LV contractility in patients with congenital heart disease, because it has been known that The LV dP/dt_max_ is not influenced by wall motion abnormalities or variations in ventricular anatomy and morphology.^[16]^ Regarding the utility of dP/dt_max_ as a bedside monitoring, it has been suggested that the Ao dP/dt_max_ is closely correlated with the LV dP/dt_max_, which means Ao dP/dt_max_ may be potential as a less invasive method to determine LV contractility especially in patients with congenital heart disease.^[5]^ Recently, Kawasaki et al^[6]^ reported that the LV dP/dt_max_ can be estimated noninvasively from brachial or radial arterial pressure waveforms using a tonometry. They also showed that dP/dt_max_ derived from peripheral arteries have significant linear relationships with Ao dP/dt_max_, despite pressure differences between the measured sites.

In infants and children, postoperative ventricular failure has been considered as a major cause of death and important predictor of clinical outcome after congenital heart surgery.^[17–19]^ At the time of completion of operation during congenital heart surgery, accurate quantification of ventricular contractility may help to decide which treatment will be effective in the critical period. Although it has been investigated the usefulness of noninvasive bedside monitoring of ventricular contractility,^[20]^ it remains uncertain to determine ventricular contractility and cardiac output in patients who have defective or distorted heart. During the cardiac surgery, transesophageal echocardiography has been most commonly used to evaluate the surgically corrected structures and function. However, echocardiographic derived parameters may be invalid resulting from abnormal geometry and load- and age-dependent properties.^[21]^ Although Doppler myocardial imaging, strain rate, and backscatter were introduced,^[22–25]^ these methods have been challenged in ensuring data standardization and inter- and intra-individual variability requiring a highly specialized echocardiographic technique.^[26]^ Particularly during the weaning period, unrecovered myocardium of small children is vulnerable to little changes in volume or pressure loading, resulting in hemodynamic instability. Additionally, Ao dP/dt_max_ can also be measured without additional invasive procedure, because we can obtain Ao pressure waveforms via the pressure monitoring line connected to Ao root cannulation of CPB circuit before discontinuation from CPB. In this regard, our study adds value in the sense that continuous measurement of dP/dt_max_ may provide beat-to-beat information about ventricular contractility.

Many types of noninvasive hemodynamic monitoring devices have been used in children, such as arterial pulse contour analysis, ultrasound, and bioreactance technique.^[27–29]^ In adults undergoing coronary artery bypass surgery, femoral dP/dt_max_ obtained from arterial waveform analysis using the transpulmonary thermodilution monitor (PiCCOplus; Pulsion, Munich, Germany) has shown to be closely related to LV dP/dt_max_ during inotropic administration.^[30]^ Although disappointing results have been reported following application of these noninvasive hemodynamic monitoring for neonates and small children,^[31–33]^ evidence has been growing to show the applicability of dP/dt_max_. Recently, it has been suggested that the dP/dt_max_, which is provided using a device embedded arterial pulse contour analysis (Mostcare/PRAM; Vytech, Padova, Italy) can be used as an index of ventricular contractility.^[34]^ In a study for critically ill children, the dP/dt_max_ derived from PRAM has been used as a parameter representing ventricular contractility and shown to track the change according to inotropic use.^[35]^

However, our study findings indicated a relatively low correlation coefficient between the Ao and radial dP/dt_max_. We speculated that vascular properties may be different according to measuring sites in the post-CPB period, although it remains uncertain why a central-to-peripheral arterial pressure gradient occurs after CPB.^[36,37]^ It is thought that marked arterial constriction may contribute to the damped transmission of the pressure pulse to the radial artery and intensifies discrepancy of the pressure.^[38]^ In addition, Kanazawa et al^[7]^ reported that central-to-peripheral arterial pressure gradient may be caused by the differences in pulse wave velocity and arterial elasticity between central and peripheral arteries. Even given that children may have age-related differences in vascular mechanical characteristics,^[39,40]^ systolic arterial pressure measured in peripheral artery is still displayed lower value than central arterial pressure in the weaning period.^[8]^ Collectively, the decrease in pulse wave velocity caused by decreased arterial elasticity may induce discrepancies of pressure rise and peak pressure between aortic and radial arterial waveforms related to CPB. In consequence, we can observe that the radial dP/dt_max_ was lower than the Ao dP/dt_max_, and there was a weak correlation between the 2 values immediately after CPB.

There were some limitations to this study. First, it was a retrospective study from a single medical center with a small sample size. In addition, it should be considered that patients with congenital heart disease may have varying vascular properties. It has been known that arterial elastance is decreased or increased depending on pathologies of cardiovascular anatomic lesion,^[41,42]^ and that elastic characteristics of aorta are also impaired in congenital anomaly involved aorta even after corrective surgery.^[43,44]^ In this study, we only included pediatric patients with uncomplicated congenital heart disease without significant cyanosis, and undergoing complete correction of the congenital heart defect. To extend the application of this approach, further studies are needed in children with complicated congenital heart disease such as functional single ventricle. In addition, children have age-related differences in vascular mechanical characteristics.^[40]^ Therefore, it is also needed to investigate whether radial and Ao dP/dt_max_ relationship after CPB weaning is different according to different age groups although we included patients whose ages ranged from 13 days to 6 years. Second, we could not measure simultaneously LV dP/dt_max_ via direct catheterization. Instead of direct catheterization, noninvasive method to estimate LV dP/dt_max_ using Doppler signals of mitral insufficiency has been widely used,^[45,46]^ although this measurement has some shortcomings that it can be only used in presence of mitral insufficiency. In a previous report, the radial dP/dt_max_ has been found to be closely correlated with the dP/dt_max_ derived on mitral regurgitation in patients with heart failure.^[47]^ Therefore, confirmation study is needed to validate the relationship between peripheral dP/dt_max_ and the dP/dt_max_ from Doppler measurement of mitral regurgitation during the weaning period. Third, we did not perform femoral arterial catheterization. Radial artery is the first choice of cannulation site for arterial pressure monitoring during congenital heart surgery in our institution, however, radial catheterization must be not easy to perform in small children. It has been reported that radial-to-femoral systolic pressure gradient was observed during the first 10 minutes after CPB in pediatric cardiac surgery.^[48]^ Therefore, it is expected that femoral dP/dt_max_ may more reliably reflect the dP/dt_max_ of central artery, and relationship between femoral and Ao dP/dt_max_ may be different from radial-Ao dP/dt_max_ relationship. In addition, we could not examine changes in the relationship between Ao and radial dP/dt_max_ before and after CPB, because we have recorded only radial arterial waveforms before CPB. Finally, LVEF and FS that we employed as measure of postoperative ventricular performance still may be questionable for accurate assessment of LV contractility due to their load-sensitive properties. In this respect, we thought that VIS_max_ may be a more valuable index as a postoperative outcomes reflecting ventricular function. In pediatric cardiac surgery, VIS_max_ has been known to be associated with LV systolic function, postoperative morbidity and mortality.^[49–51]^ Therefore, we suggest that monitoring of Ao dP/dt_max_ may be useful to predict greater requirement of vasoactive inotropic drugs, reflecting early postoperative ventricular function.

## 5 Conclusions

Immediately after children are weaned from CPB, the radial dP/dt_max_ was weakly correlated with Ao dP/dt_max_. However, the radial dP/dt_max_ was not interchangeable with Ao dP/dt_max_, because vascular properties might be changed differently between central and peripheral arteries, resulting in a disparity of pressure waveforms. The Ao dP/dt_max_ was still closely associated with early postoperative ventricular function in congenital heart surgery. Therefore, intraoperative measurement of Ao dP/dt_max_ via aortic cannula may provide information about beat-to-beat ventricular contractility during immediate post-CPB period. However, further studies are needed for patients with complicated congenital heart disease including functional single ventricle, and for different age groups to extend the usefulness of Ao dP/dt_max_ to predict postoperative ventricular function.
